# Prostate Cancer Chemoprevention Targeting Men with High-Grade Prostatic Intraepithelial Neoplasia (HGPIN) and Atypical Small Acinar Proliferation (ASAP): Model for Trial Design and Outcome Measures

**DOI:** 10.4172/jctr.1000105

**Published:** 2012-01-21

**Authors:** Nagi Kumar, Theresa Crocker, Tiffany Smith, Shahnjayla Connors, Julio Pow-Sang, Philippe E. Spiess, Kathleen Egan, Gwen Quinn, Michael Schell, Said Sebti, Aslam Kazi, Tian Chuang, Raoul Salup, Mohamed Helal, Gregory Zagaja, Edouard Trabulsi, Jerry McLarty, Tajammul Fazili, Christopher R. Williams, Fred Schreiber, Kyle Anderson

**Affiliations:** 1 Departments of Epidemiology, Health Outcomes and Behavior, Biostatistics, H. Lee Moffitt Cancer Center & Research Institute, University of South Florida College of Medicine, Florida, USA; 2 Oncological Sciences, University of South Florida College of Medicine, Tampa Florida; 3 James A. Haley V.A. Hospital, Tampa, FL; 4 University of Chicago, Chicago, IL; 5 Jefferson Medical College, Philadelphia, PA; 6 LSUHSC – Feist-Weiller Cancer Center, Shreveport, LA; 7 Overton Brooks VA, Shreveport, LA; 8 University of Florida – Jacksonville; 9 Watson Clinic - Center for Cancer Care & Research, Lakeland, FL; 10 Minneapolis VA Medical Center, Minneapolis, MN

**Keywords:** Prostate cancer, Chemoprevention, Green tea polyphenols, HGPIN, ASAP, PSA, Steroid hormones, Proliferative and apoptotic markers, Proteasome inhibition

## Abstract

In spite of the large number of nutrient-derived agents demonstrating promise as potential chemopreventive agents, most have failed to prove effectiveness in clinical trials. Critical requirements for moving nutrient-derived agents to recommendation for clinical use include adopting a systematic, molecular-mechanism based approach and utilizing the same ethical and rigorous methods such as are used to evaluate other pharmacological agents. Preliminary data on a mechanistic rationale for chemoprevention activity as observed from epidemiological, *in vitro* and preclinical studies, phase I data of safety in suitable cohorts, duration of intervention based on time to progression of preneoplastic disease to cancer and the use of a valid panel of biomarkers representing the hypothesized carcinogenesis pathway for measuring efficacy must inform the design of phase II clinical trials. The goal of this paper is to provide a model for evaluating a well characterized agent- Polyphenon E- in a phase II clinical trial of prostate cancer chemoprevention.

## The Disease: Prostate Cancer

The American Cancer Society estimates that there will be 240,890 new cases of prostate cancer (CaP) in the United States (US) in 2011, and 33,720 men will die from this disease [1]. The initiation and progression of CaP involves a complex array of both exogenous and endogenous factors [2-5]. In prostate epithelial tissues, genetic progression and loss of cellular control functions are observed as the cell and tissue phenotype changes from normal to dysplasia (prostatic intraepithelial neoplasia or PIN), then to increasingly severe dysplasia (high grade PIN or HGPIN), to superficial cancers and finally to invasive disease [3]. Although it is clear that clinical CaP incidence and mortality vary greatly between populations, the frequency of latent CaP is evenly distributed among populations, suggesting that external factors such as diet, physical activity and other lifestyle factors are important in the transformation from latent into more aggressive, clinical cancer [2-5]. The features of prostate cancer, namely high prevalence, long latency, significant mortality and morbidity, and the availability of HGPIN and ASAP as intermediate predictive stages of progression, provide the most promise for evaluating agents for chemoprevention [6-9].

Studies indicate that HGPIN is the primary premalignant lesion of CaP [3,10-13] and it is therefore considered a possible pre-invasive precursor of CaP [3]. More recently, atypical small acinar proliferation (ASAP), characterized by a focus of glands that do not contain sufficient cytologic or architectural atypia to establish a definitive diagnosis of cancer [4,14], has emerged as a diagnosis of exclusion but with a greater association to prostatic carcinoma than HGPIN. Both HGPIN and ASAP are associated with progressive abnormalities of phenotype and genotype, which are intermediate between normal prostatic epithelium and cancer, indicating impairment of cell differentiation and regulatory control with advancing stages of prostatic carcinogenesis.

## Prostate Cancer Chemoprevention

Chemoprevention refers to the inhibition of preinvasive and invasive cancer and its progression or treatments of identifiable precancers [8,15]. Chemoprevention efforts require a thorough understanding of the mechanism of carcinogenesis including signaling and metabolic pathways and genetic progression pathways. New technologies in genomics and proteomics have spurred this field of research. The use of this knowledge to develop pharmacologic agents (including nutrient-derived) to reverse or halt the process of carcinogenesis is called chemoprevention. Agents for chemoprevention include anti-promotion and anti-progression agents that prevent the growth and survival of cells that are already committed to become malignant [8,15]. Several nutrient-derived agents have demonstrated promise as potential chemopreventive agents in the prevention of prostate cancer. The goal of this paper is to provide a model for utilizing a systematic approach in planning and evaluating a well characterized agent- Polyphenon E in a phase II clinical trial for prostate cancer chemoprevention.

## Promising Agent for Chemoprevention of Prostate Cancer: Green Tea Polyphenols (GTP)

The use of green tea for prostate cancer chemoprevention has been the focus of many laboratory and clinical studies. The chemopreventive actions of green tea are attributed to the presence of green tea catechins (GTCs), especially epigallocatechin gallate (EGCG) [16]. Clinical studies suggest that GTCs are effective in the chemoprevention of human prostate cancer [17-19]. This prior data justifies the rationale for men with HGPIN and ASAP as a target high-risk population for evaluating promising chemopreventive agents for the prevention of prostate cancer, as we have in this ongoing phase II clinical trial of Polyphenon E.

## Agent Selection and Description

EGCG is the major and most active catechin in green tea and is the most commonly studied GTC *in vitro*, because of its relative abundance in green tea extracts and strong cancer preventative properties [20-22]. However, EGCG has low rates of absorption and bioavailability when administered orally [23-25] and studies indicate that whole mixtures of GTCs may more accurately reflect the human consumption of green tea. This is possibly due to the fact that tea constituents other than catechins may also have anti-carcinogenetic activity and the combined interaction of tea components and catechins may contribute to the effectiveness of the anticarcinogenic activities of GTC mixtures [25-29]. The lack of assurance of infusion contents, differences in tea origin and brewing techniques, all which affect the tea catechin content, have made it necessary to use more standardized GTC mixtures for clinical purposes [30].

Based on the safety profile established in phase I clinical trials [24,31,32], we selected a standardized decaffeinated botanical formulation of green tea catechins, Polyphenon E (Mitsui Norin Co., Ltd., Shizuoka, Japan; Mitsui Norin) for use in this clinical trial. Polyphenon E, is a botanical drug substance containing a mixture of catechins originating from the leaves of green tea (Camellia sinensis). The final product contains 85-95% total catechins; the main component is EGCG, which comprises 56-72% of the material. Other catechins present in Polyphenon E include EC, ECG, EGC, GCG, GC, CG and catechin. Polyphenon E contains minimal amounts of caffeine (<1.0%) and may also contain small quantities of theobromine (<1.0%) and gallic acid (<0.5%). The investigational product used in this study is a hard gelatin capsule containing enough Polyphenon E to deliver 200 mg EGCG per capsule. Placebo capsules are hard gelatin capsules containing pregelatinized starch, microcrystalline cellulose, colloidal silicon dioxide, and magnesium stearate. Both are manufactured under contract to NCI, DCP in compliance with current good manufacturing practice regulations and packaged in 150 cc white HDPE bottles, with 100 capsules per bottle. An investigator-initiated IND was obtained for this agent at this dose and for this indication.

## Hypothesis and Objectives

Our broad and long-term goal is to develop safe, non-toxic agents that can be consumed safely over long periods to prevent progression in men at high risk of prostate cancer using validated markers relevant to prostate carcinogenesis and to monitor changes in disease incidence. Based on the strong preliminary evidence from epidemiological, preclinical, laboratory and early phase I and II pilot studies, the central hypothesis for this Phase II clinical trial is that men with HGPIN or ASAP who receive Polyphenon E at a dose of 400 mg EGCG per day for 12 months will have significantly decreased progression of HGPIN or ASAP to prostate cancer compared with men with HGPIN or ASAP who take placebo.

**Figure 1:**
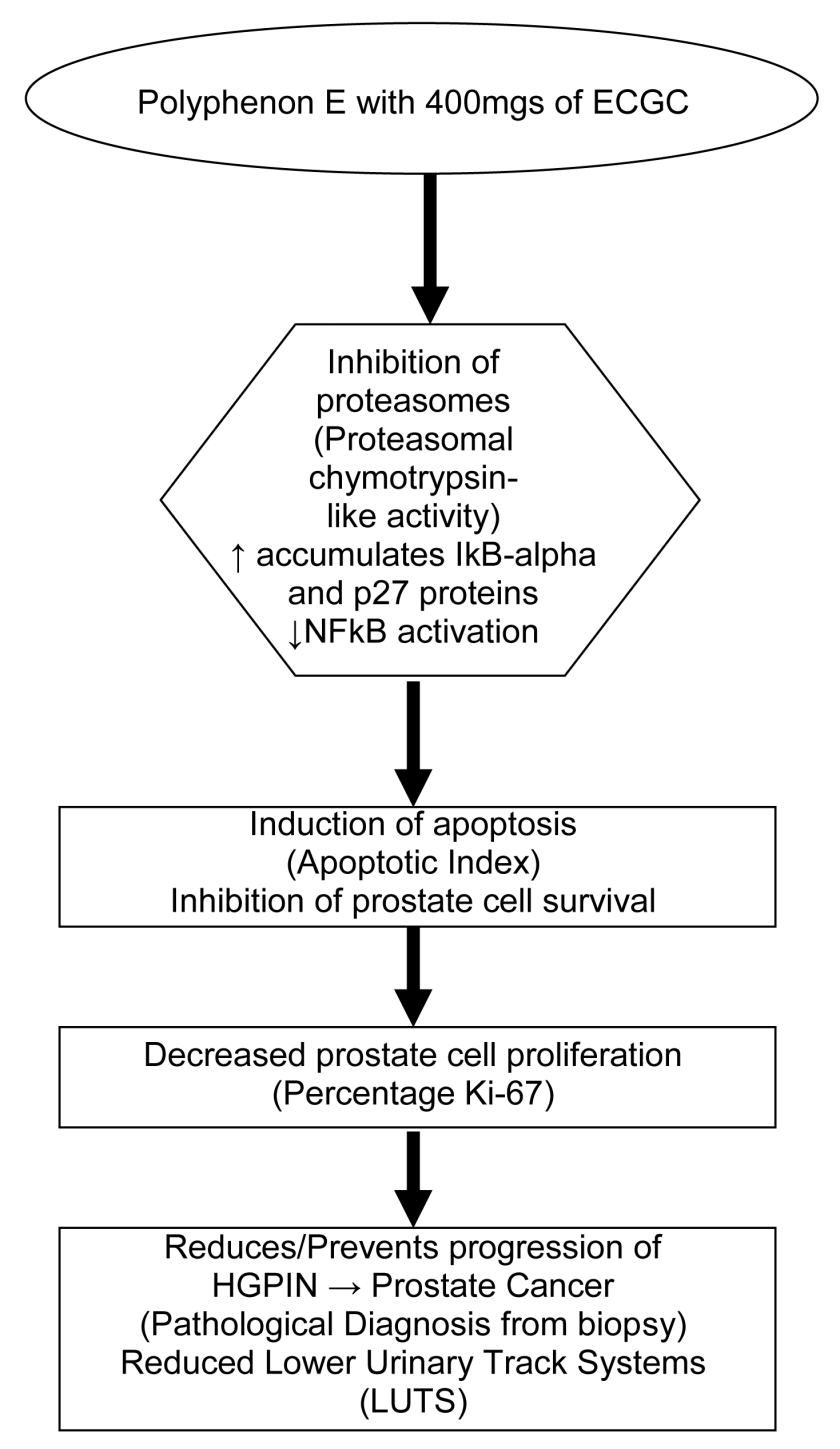
Primary Mechanism of Green Tea Polyphenons (Polyphenon E) in Prostate Carcinogenesis.

GTCs have been shown to exert anticancer activities through a variety of different mechanisms [9,30,33-35]. We have proposed a novel cumulative model in which six major mechanisms of GTP chemoprevention (Figure 1): proteasome inhibition, cell cycle arrest, inhibition of cell proliferation, apoptosis, suppression of progression and inhibition of metastasis, work sequentially and in concert through the NFκB pathway to exert chemopreventive action on prostate cancer cells [36]. We hypothesize that the primary pathway by which GTCs in Polyphenon E, specifically EGCG, will induce prostate epithelial cell apoptosis, is via the proteasome inhibition pathway (accumulating IκBα and p27 proteins, decreasing NFκB activation), resulting in inhibition of prostate cell survival and induction of apoptosis, thereby decreasing progression from HGPIN or ASAP to prostate cancer.

## Secondary Study Objectives

The primary objectives of this study are to evaluate the effectiveness and safety of Polyphenon E (200 mg EGCG BID) (vs. placebo) administered for 1 year to men, following the diagnosis of HGPIN and/or ASAP. The primary efficacy endpoint is the progression to prostate cancer at one year. Men will be examined at three (ASAP) and six months (both) for a rise in PSA, as defined by a re-confirmed PSA increase of >0.75ng/ml or the development of a prostate nodule, and biopsied at 3 or 6 months if a rise in PSA or a nodule is detected, or after 12 months and the frequency of HGPIN, ASAP and CaP defined. If prostate cancer develops during the course of investigation, we will compare the extent and grade of the cancer following treatment. The primary safety endpoint is the incidence and severity of AE's during intervention evaluated using standard assessment of toxicity (ver. CTCAE 3.0) [37] based upon detailed questionnaires, including symptoms, clinical evaluation, and assessment of biological samples (comprehensive metabolic panel (CMP), complete blood count (CBC) and LFTs (SGOT/SGPT) completed by Quest Diagnostic laboratories throughout the study. Compliance to intervention is monitored using pill counts and agent logs, quarterly diet diaries and plasma catechins using HPLC-EC at baseline, 6 and 12 months. The effect of treatment with Polyphenon E on lower urinary tract symptoms using the Lower Urinary Tract symptoms Scale [38] and quality of life (QOL) using Rand Short-form (SF)-36 [39] is also evaluated.

Secondary objectives include an exploration of the fundamental molecular pathways that contribute to the chemopreventive activity of Polyphenon E in the prostate. The goal is to determine whether Polyphenon E (200 mg EGCG BID) (vs. placebo) administered for one year results in inhibition of proteasome activity, suppression of cell proliferation and induction of apoptosis in prostate tissue biopsies. In addition, we will explore other plausible pathways by which Polyphenon E may reduce carcinogenesis including stabilization and accumulation of the tumor suppressor p53 and the pro-apoptotic protein Bax, and the inhibition of VEGF (with inhibition of angiogenesis), and MMP-2 and MMP-9 (markers of angiogenesis which mediate invasion and metastasis) [40]. In addition to developing and refining the fundamental pathways of Polyphenon E, these exploratory studies have the potential to define surrogate endpoints for cancer incidence in the prostate.

## Study Design

This 5-year project is a randomized double-blind placebo controlled phase II clinical trial examining the safety and efficacy of Polyphenon E at a dose of 200 mg EGCG bid administered for 12 months versus placebo on the occurrence of cancer in men with a diagnosis of HGPIN or ASAP. Cohort participants are recruited from nine research sites in the United States. We have established special teams for administration, recruitment, intervention and retention, biomarkers and data safety monitoring who work closely to provide efficient, standardized and centralized services for successful implementation of this proposal. In addition to disease progression as the ultimate outcome in this study, we will also evaluate a selected combination of biochemical (PSA, steroid hormones), morphological (cytopathology, Ki-67) and apoptotic index- intermediate endpoint biomarkers relevant to prostate carcinogenesis. We have also included markers of quality of life and exploratory studies examining the molecular targets of Polyphenon E.

## Screening, Registration and Run-in Procedures

The subjects for this study are recruited primarily from Urology and Urology Oncology clinical practices as approved by Institutional Review Boards at each site. The study population consists of men who meet the following eligibility criteria: (a) ages 30-80; (b) diagnosis of HGPIN and/or ASAP within 6 months; (c) PSA≤ 10 ng/ml; (d) No history of cancer except for non-melanoma skin cancer; (e) No known history of hepatic or renal disease (LFTs (SGOT/SGPT) < 2.5 × upper limit of normal, Actual creatinine clearance of >60 utilizing the Cockroft-Gault formula (1976); (f) men who do not or can refrain from drinking six servings of hot tea or 12 servings of iced tea per week or taking green tea supplements; (g) not taking steroid hormones or medications which have known impact on PSA, and; (h) ECOG performance status of 0-1. Eligible subjects are invited to participate and begin screening and run-in procedures. Informed consent is obtained prior to any data collection. Data collected at screening/pre-randomization include lab values for prostate specific antigen (PSA), comprehensive metabolic panel (CMP), complete blood count (CBC), direct bilirubin, prothrombin time/partial thromboplastin time (PT/PTT) and lactate dehydrogenase (LDH). Medical history and concomitant medications are verified and subjects complete a demographic questionnaire. For the run-in period, subjects are instructed on completion of the study agent intake log and the 2-day diet recall with forms provided. A 10 day supply of multivitamin/mineral supplement is provided and subjects are instructed to take once daily until they return to the clinic. The screening and run-in period takes place over a 7-10 day period and is designed to assure ability to maintain compliance with agent intake and required study logs.

## Randomization

Final eligibility is confirmed based on the screening tests and compliance during the run-in period (multivitamin/mineral intake and completion of required study logs). Baseline lower urinary tract symptoms (LUTS) and quality of life (QOL) questionnaires are completed; 2-day diet recall forms are collected and reviewed; signs/symptom assessment is completed and concomitant medications are reviewed. Serum and plasma samples are collected for baseline diagnostic marker analysis, baseline catechin measurements and banking. Urine is collected for baseline diagnostic marker analysis and tissue from diagnostic biopsy is collected for baseline measurements and banking.

Randomization (Polyphenon E, 200 mg EGCG bid or placebo) occurs once a subject is confirmed to meet all eligibility requirements using the SRAR system. SRAR is a web delivered application that records subject registrations and provides blocked randomization assignments using randomization lists developed by our biostatistics department to stratify by diagnosis (HGPIN or ASAP) and by recruitment site, and to ensure that a corresponding placebo and treatment assignment is planned for each strata. Users are securely logged in and then enter subject's registration information. A number corresponding with one of two arms is assigned and an email is sent to the site trial coordinator (blinded) and pharmacy designee (unblinded) stating the treatment/randomization assigned to the subject. As this is a double-blinded study the assignment is hidden from both the subject and investigators.

## Adherence/Compliance during Intervention and Treatment

During study participation, instructions are provided to self administer one capsule of study agent daily each morning and evening, with meals. On the day of the biopsy, subjects are instructed to take the pill with lunch if they have an afternoon appointment. In addition to taking the study agent, subjects are instructed to continue to take one multivitamin/mineral daily. Subjects are also asked to: complete diet recall forms at specified time periods, limit consumption of tea and foods containing high levels of catechins, avoid herbs and nutritional supplements containing catechins, complete daily study agent intake logs, record concomitant medications, and any signs or symptoms. The goal is to maintain ≥85% compliance with study agent intake. Subjects return for monthly visits for the next twelve months.

## Pathological Review

To assure inter-tester reliability of diagnosis of HGPIN (exclusive) or ASAP at all sites, in addition to using one standard diagnosis criteria, at various time points slides from each coordinating site are forwarded for review by the sponsoring site pathologist and the diagnosis verified for consistency. At points of discrepancy between the two pathologists, a third neutral senior pathologist reviews the slide and provides his finding. In addition, quality control studies to confirm diagnosis of HGPIN are done on samples from each site using the PIN-4 stain protocol.

## Monitoring

All subjects are assessed clinically for toxicities during screening (prior to randomization), monthly during intervention, and at the end of intervention. All monthly visits include lab work for safety monitoring (hepatic function panel, PT/PTT and LDH). In addition to regular monthly labs, CBC, CMP, direct bilirubin and PSA are completed at months 3 (PSA only for men with ASAP), 6 and 12 or end of treatment. Compliance with study agent intake is measured during monthly safety checks via pill counts. Study agent intake logs are used to assess signs/symptoms and concomitant medications. Any toxicities (adverse events) occurring during the investigation are reviewed by the treating physician and managed according to standard medical practice.

Clinical procedures are as follows: DRE (month 3 -ASAP; month 6 and 12-all); repeat biopsy (month 3-if palpable prostate nodule or confirmed rise in PSA-ASAP; month 6-ASAP or palpable prostate nodule or confirmed rise in PSA-HGPIN; month 12-all); physical exam (month 12). Additionally, the following data or samples are collected at varying time points: 2-day diet recall, LUTS and QOL (months 3, 6, 12); Serum and urine for diagnostic marker analyses; plasma for catechin measurements; serum for banking (months 6 and 12). Seven+- 3 days post-treatment with study agent, telephone contact is made to assess signs/symptoms and concomitant medications during this time. Study participation is considered complete following this contact.

## Treatment Interruption or Termination

Rules for dose suspension and discontinuation are determined by the type (related to liver function or other) and grade (as determined by CTCAE Term v 3.0) of adverse event. Per FDA requirement, following any recurrent grade 1 LFT elevation (second occurrence of the same elevation) study drug is withheld for at least one week and liver function is monitored until resolution to normal. Following any LFT elevation grade ≥2, study drug is permanently discontinued and liver function monitored until resolution to normal. Study drug is also permanently discontinued for grade 3 and 4 AEs, unless clearly not related to therapy. There are no reductions in the Polyphenon E dose; if AEs occur that require suspension of drug administration, the dose remains the same once treatment resumes. If a subject has an unresolved AE at the time of withdrawal from study treatment, he will be followed until resolution of the event, if possible. If an AE persists for more than 30 days after a subject goes of agent, the subject will be referred to his personal physician.

## Statistical Analysis

The original assumptions for the statistical power calculations for this study were derived from a preliminary study by Dr. Bettuzzi et al. [18]. Based on recent studies, with ASAP gaining ground as a separate diagnosis, one-year progression rates for exclusive HGPIN range from 4.5% to 27%, centering around 20%. Meanwhile one-year progression rate estimates to prostate cancer for men with ASAP range from 25% to 59%, centering around 40%. Since we anticipate a 50-50 mixture of these two diagnoses, the estimated overall one-year progression rate for this study is 30%. To be conservative, the sample size is chosen to achieve 80% power if the treatment reduces the rate of prostate cancer within one year from 30% to 15%, a 15% reduction compared to the 27% decrease (from 30% to 3%) observed by Dr. Bettuzzi et al. [18]. The use of blocked randomization with diagnosis being one of two strata will ensure a balance of diagnoses in the two study arms. The analysis will be done overall using a single 2×2 unconditional test.

We plan to perform an interim analysis of the primary endpoint. The α level for the interim look will be .0125, leaving α = .041 for the final analysis. For an unconditional test at the two-sided 0.041 significance level, this requires 123 per group, for a total of 246. The interim look will be made when the first third of the patients (40 per group) has been on study for one year, which is when the primary endpoint is resolved for these patients. This is expected to occur at about the half-way time point in the study. The power for early termination will be 19% if the true cancer rates are 30% and 15%. If the true cancer rates are the same as the sample estimates from the Bettuzzi study, though, the power of early termination will be 85%. Thus, this interim analysis helps to guard against large but conceivable differences in cancer rates between the treatment arms.

In order to achieve 240 evaluable subjects, we will accrue a total of 272 subjects, which allows for an estimated dropout rate of 10% during the run-in phase prior to randomization. This will provide us with 80% power for rates of 30% vs. 15% and 98% power if those rates are 30% and 10%. We also have 87% power for rates of 25% vs. 10%, and 85% power for rates of 20% vs. 7% and 85% power for rates of 15% vs. 4%. With the current sample size, the study will have greater power to detect a 15% difference even where the control group rates will be lower than 30%. In order to assess the effect of treatment on the extent of prostate cancer in those subjects who do develop prostate cancer while on study, we will use the following Gleason grade categories: 0 (for all subjects not diagnosed with prostate cancer), 1–6, 7–8, 9–10, and apply a Tukey trend test at α = .05 to the 2×4 contingency table.

## Data Management and Study Monitoring

All collected data is entered from source documents or case report forms (CRF's) directly into the web-based ONCORE system by authorized, trained staff. Toxicities are monitored continuously through the trial by the PI and study physician at each site. Additionally, the study team is guided by an External Data and Safety Monitoring Board (EDSMB). Statistical analyses were performed and reviewed by the EDSMB when fifty patients completed at least one month on study and are repeated every six months (and more often if needed). The EDSMB can recommend early termination if a serious imbalance occurs and the estimated risk of harm appeared to warrant such action. Additionally, the study is monitored following a monitoring plan developed by the Protocol Review and Monitoring System at Moffitt Cancer Center.

## Future Directions

Poly E in HGPIN and ASAP is a Phase II, Randomized, Double-blind, Multi-centered Study, are ongoing at several clinical sites across the United States. The goal of this study is to evaluate the efficacy and safety of a promising chemopreventive agent (Polyphenon E) in a high risk population (men with HGPIN and/or ASAP) to inform the design of a larger Phase III clinical trial. Demonstration of efficacy and safety of Polyphenon E will be an important outcome, even if the mechanism(s) involved is not identified in this study, or is shown to be different from the one proposed. With baseline and post intervention specimens available including tissue from biopsy, serum, buffy coat and urine banked, we have the opportunity to explore and validate alternative and evolving IEBs as well as mechanisms. The systematic approach presented here may serve as a model for the evaluation of other chemopreventive agents for prostate cancer and other solid organ tumor types.

## Figures and Tables

**Figure 2:**
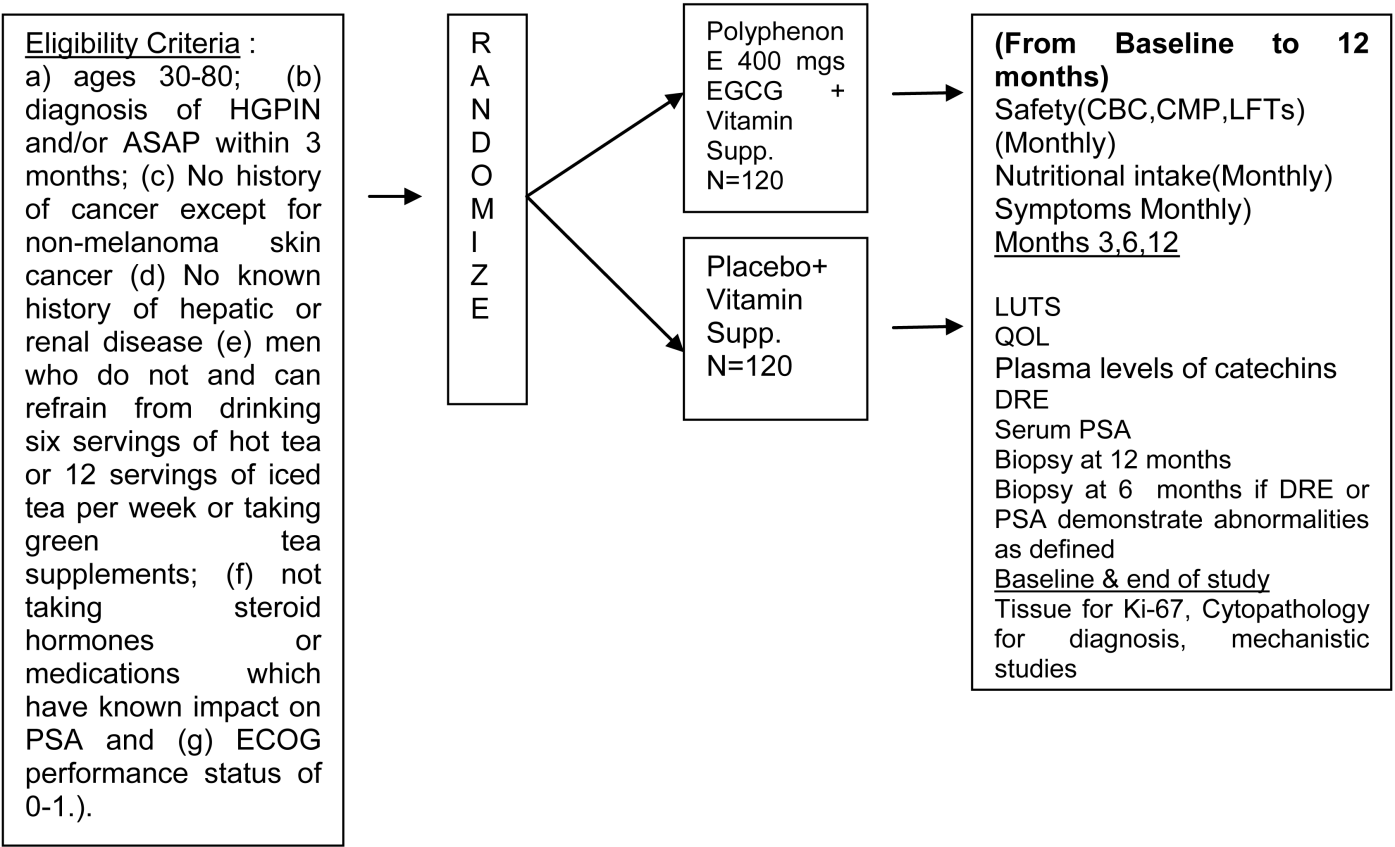
Study Schema for a Phase II clinical trial of Polyphenon E in Prostate Cancer Chemoprevention targeting High-grade Prostatic Intraepithelial Neoplasia (HGPIN) and Atypical Small Acinar Proliferation (ASAP).

**Table 1:** Clinical evaluations and procedures.

Evaluation/Procedure	Screening/Pre-Randomization	Run-in	Baseline/Random-ization	Months 1–11	Month 3	Month 6	Month 12 or Early Termination	Post-Tx Day 7 Phone Contact
Informed Consent Signed/Registration Number Assigned	▪							
Demographic Questionnaire	▪							
Physical Exam (weight, height and vital signs, hip circumference measurement)	▪						▪	
AEs (Signs and Symptoms)	▪	▪	▪	▪	▪	▪	▪	▪
Concomitant Medications	▪	▪	▪	▪	▪	▪	▪	▪
PSA	▪^1^				▪^3^	▪	▪	
DRE	▪^1^				▪^3^	▪	▪	
Serum Chemistry and Hematology (CMP, CBC)	▪				▪	▪	▪	
Hepatic Function Panel^2^, LDH, PT/PTT	▪			▪	▪	▪	▪	
Pathology – Biopsy (12 core biopsies)	▪^3^					▪^4^	▪	
Dispense Multivitamin/Mineral Supplement	▪		▪	▪	▪	▪		
Collect 2-day Diet Recall Forms^5^			▪		▪	▪	▪	
Compliance Check (review log and perform pill count)			▪	▪	▪	▪	▪	
Randomization			▪^6^					
Collect Serum for Banking			▪			▪	▪	
Request Biopsy Specimen for Endpoint Measurements and Banking			▪				▪	
Plasma Catechin Levels			▪			▪	▪	
Diagnostic Markers^7^			▪			▪	▪	
LUTS			▪		▪	▪	▪	
QOL^8^			▪		▪	▪	▪	
Dispense Study Agent			▪^9^	▪^9^	▪^9^	▪^9^		

1 DRE does not need to be performed at the screening/pre-randomization visit if results are available from TRUS performed prior to diagnostic biopsy and within six months of randomization.

2 Includes albumin, direct and total bilirubin, alkaline phosphatase, ALT, AST, total protein.

3 Results from the diagnostic biopsy will be used to confirm presence of HGPIN or ASAP for eligibility; if the subject is randomized, slides from the diagnostic biopsy will be obtained and used for pre-intervention histopathology drug effect measurements.

4 Men will be re-biopsied at six months (if enrolled with ASAP) or if PSA increases >0.75 ng/ml or development of a palpable prostate nodule is observed in men with HGPIN. Considering the normal day to day variance of PSA, any rise in PSA will be confirmed with a second test prior to repeat biopsy at 6 months (HGPIN). If biopsy is performed, samples for endpoint analysis and banking will be requested.

5 Dietary recall forms and instructions will be distributed at the screening and month 2, 5 and 11 clinic visit to be completed by the subject prior to the next scheduled visit.

6 Subjects should not be randomized until eligibility has been confirmed; if the subject does not meet eligibility criteria, he should be taken off study.

7 Prostate cancer-associated diagnostic marker-1 (PCADM-1) level in serum; HGPIN-specific marker ABCA5 in urine. Urine for ABCA5 should be collected as the first void urine at the site visit, prior to any manipulations (*e.g.*, DRE).

8 Rand Short-form (SF)-36 (Medical Outcomes Study SF36).

9 One bottle (100 capsules) of study drug will be dispensed after randomization. Additional bottles will be dispensed as needed at monthly clinic visits to ensure that the subject has enough study drug to last until the next clinic visit.

10 Please note that PSA is only required at month 3 for those men enrolled with ASAP.

11 Please note that DRE is only required at month 3 for those men enrolled with ASAP.

## References

[1] Bostwick DG, Burke HB, Djakiew D, Euling S, Ho SM (2004). Human prostate cancer risk factors. Cancer.

[2] Mohamed MA, Greif PA, Diamond J, Sharaf O, Maxwell P (2007). Epigenetic events, remodelling enzymes and their relationship to chromatin organization in prostatic intraepithelial neoplasia and prostatic adenocarcinoma. BJU Int.

[3] Bostwick DG, Qian J (2004). High-grade prostatic intraepithelial neoplasia. Mod Pathol.

[4] Epstein JI, Herawi M (2006). Prostate needle biopsies containing prostatic intraepithelial neoplasia or atypical foci suspicious for carcinoma: implications for patient care. J Urol.

[5] Burzon D, Kahnoski RJ, Bennett JK (2005). Men with high-grade prostatic intraepithelial neoplasia (HGPIN) remain at high risk for prostate cancer regardless of whether HGPIN is detected on subsequent giopsies. Urology.

[6] Greenwald P, Kelloff GJ (1996). The role of chemoprevention in cancer control. IARC Sci Publ.

[7] Kelloff GJ, Lieberman R, Steele VE, Boone CW, Lubet RA (2001). Agents, biomarkers, and cohorts for chemopreventive agent development in prostate cancer. Urology.

[8] Kelloff GJ, Lieberman R, Steele VE, Boone CW, Lubet RA (1999). Chemoprevention of prostate cancer: concepts and strategies. Eur Urol.

[9] Khan N, Adhami VM, Mukhtar H (2009). Review: green tea polyphenols in chemoprevention of prostate cancer: preclinical and clinical studies. Nutr Cancer.

[10] Gokden N, Roehl KA, Catalona WJ, Humphrey PA (2005). High-grade prostatic intraepithelial neoplasia in needle biopsy as risk factor for detection of adenocarcinoma: current level of risk in screening population. Urology.

[11] Kronz JD, Allan CH, Shaikh AA, Epstein JI (2001). Predicting cancer following a diagnosis of high-grade prostatic intraepithelial neoplasia on needle biopsy: data on men with more than one follow-up biopsy. Am J Surg Pathol.

[12] Naya Y, Ayala AG, Tamboli P, Babaian RJ (2004). Can the number of cores with high-grade prostate intraepithelial neoplasia predict cancer in men who undergo repeat biopsy?. Urology.

[13] San Francisco IF, Olumi AF, Kao J, Rosen S, DeWolf WC (2003). Clinical management of prostatic intraepithelial neoplasia as diagnosed by extended needle biopsies. BJU Int.

[14] Iczkowski KA (2006). Current prostate biopsy interpretation: criteria for cancer, atypical small acinar proliferation, high-grade prostatic intraepithelial neoplasia, and use of immunostains. Arch Pathol Lab Med.

[15] http://www.cancer.org/Cancer/ProstateCancer/DetailedGuide/prostate-cancer-key-statistics.

[16] Liao S, Kao YH, Hiipalla RA (2001). Green tea: biochemical and biological basis for health benefits. Vitam Horm.

[17] Brausi M, Rizzi F, Bettuzi S (2008). Chemoprevention of human prostate cancer by green tea catechins: two years later. A follow-up update. Eur Urol.

[18] Bettuzzi S, Brausi M, Rizzi F, Castagnetti G, Peracchia G (2006). Chemoprevention of human prostate cancer by oral administration of green tea catechins in volunteers with high-grade prostate intraepithelial neoplasia: a preliminary report from a one-year proof-of-principle study. Cancer Res.

[19] McLarty J, Bigelow RL, Smith M, Elmajian D, Ankem M (2009). Tea polyphenols decrease serum levels of prostate-specific antigen, hepatocyte growth factor, and vascular endothelial growth factor in prostate cancer patients and inhibit production of hepatocyte growth factor and vascular endothelial growth factor in vitro. Cancer Prev Res (Phila).

[20] (1996). Clinical development plan: Tea extracts, green tea polyphenols, epigallocatechin gallate. J Cell Biochem Suppl.

[21] Balentine DA, Wiseman SA, Bouwens LC (1997). The chemistry of tea flavonoids. Crit Rev Food Sci Nutr.

[22] Fujiki H (1999). Two stages of cancer prevention with green tea. J Cancer Res Clin Oncol.

[23] Chen L, Lee MJ, Li H, Yang CS (1997). Absorption, distribution, elimination of tea polyphenols in rats. Drug Metab Dispos.

[24] Chow HH, Cai Y, Alberts DS, Hakim I, Dorr R (2001). Phase I pharmacokinetic study of tea polyphenols following single-dose administration of epigallocatechin gallate and polyphenon E. Cancer Epidemiol Biomarkers Prev.

[25] Lambert JD, Yang CS (2003). Cancer chemopreventive activity and bioavailability of tea and tea polyphenols. Mutat Res.

[26] Paschka AG, Butler R, Young CY (1998). Induction of apoptosis in prostate cancer cell lines by the green tea component, (-)-epigallocatechin-3-gallate. Cancer Lett.

[27] Suganuma M, Okabe S, Sueoka N, Sueoka E, Matsuyama S (1999). Green tea and cancer chemoprevention. Mutation research.

[28] Zhou JR, Yu L, Zhong Y, Blackburn GL (2003). Soy phytochemicals and tea bioactive components synergistically inhibit androgen-sensitive human prostate tumors in mice. J Nutr.

[29] Kumar N, Titus-Ernstoff L, Newcomb PA, Trentham-Dietz A, Anic G (2009). Tea consumption and risk of breast cancer. Cancer Epidemiol Biomarkers Prev.

[30] Johnson JJ, Bailey HH, Mukhtar H (2010). Green tea polyphenols for prostate cancer chemoprevention: a translational perspective. Phytomedicine.

[31] Chow HH, Cai Y, Hakim IA, Crowell JA, Shahi F (2003). Pharmacokinetics and safety of green tea polyphenols after multiple-dose administration of epigallocatechin gallate and polyphenon E in healthy individuals. Clin Cancer Res.

[32] Chow HH, Hakim IA, Vining DR, Crowell JA, Ranger-Moore J (2005). Effects of dosing condition on the oral bioavailability of green tea catechins after single-dose administration of Polyphenon E in healthy individuals. Clin Cancer Res.

[33] Adhami VM, Ahmad N, Mukhtar H (2003). Molecular targets for green tea in prostate cancer prevention. J Nutr.

[34] Stuart EC, Scandlyn MJ, Rosengren RJ (2006). Role of epigallocatechin gallate (EGCG) in the treatment of breast and prostate cancer. Life Sci.

[35] Pandey M, Gupta S (2009). Green tea and prostate cancer: from bench to clinic. Front Biosci.

[36] Connors SK, Chornokur G, Kumar NB (2011). New Insights into the Mechanisms of Green Tea Catechins in the Chemoprevention of Prostate Cancer. Nutr Cancer.

[37] http://ctep.cancer.gov/protocoldevelopment/ectronic_applications/docs/ctcaev3.pdf.

[38] Katz G, Rodriguez R (2001). Use of a modified American Urological Association Symptom Score for the evaluation of the quality of life of patients with prostate cancer. Urology.

[39] Garratt AM, Ruta DA, Abdalla MI, Buckingham JK, Russell IT (1993). The SF36 health survey questionnaire: an outcome measure suitable for routine use within the NHS?. BMJ.

[40] Smith DM, Wang Z, Kazi A, Li LH, Chan TH (2002). Synthetic analogs of green tea polyphenols as proteasome inhibitors. Mol Med.

